# HIVE-Hexagon: High-Performance, Parallelized Sequence Alignment for Next-Generation Sequencing Data Analysis

**DOI:** 10.1371/journal.pone.0099033

**Published:** 2014-06-11

**Authors:** Luis Santana-Quintero, Hayley Dingerdissen, Jean Thierry-Mieg, Raja Mazumder, Vahan Simonyan

**Affiliations:** 1 Center for Biologics Evaluation and Research, US Food and Drug Administration, Rockville, Maryland, United States of America; 2 Department of Biochemistry and Molecular Biology, George Washington University Medical Center, Washington, DC, United States of America; 3 National Center for Biotechnology Information, U.S. National Library of Medicine, National Institutes of Health, Bethesda, Maryland, United States of America; Natural History Museum of Denmark, Denmark

## Abstract

Due to the size of Next-Generation Sequencing data, the computational challenge of sequence alignment has been vast. Inexact alignments can take up to 90% of total CPU time in bioinformatics pipelines. High-performance Integrated Virtual Environment (HIVE), a cloud-based environment optimized for storage and analysis of extra-large data, presents an algorithmic solution: the HIVE-hexagon DNA sequence aligner. HIVE-hexagon implements novel approaches to exploit both characteristics of sequence space and CPU, RAM and Input/Output (I/O) architecture to quickly compute accurate alignments. Key components of HIVE-hexagon include non-redundification and sorting of sequences; floating diagonals of linearized dynamic programming matrices; and consideration of cross-similarity to minimize computations.

**Availability:**

https://hive.biochemistry.gwu.edu/hive/

## Introduction

Sequence alignment is the critical first step of sequence analysis [1], [2], after which the alignment results are used as a source of data for numerous downstream analyses (e.g., the genetic content of short reads, pathway analysis, and etc). Before proceeding to the description of the optimized, ultra-fast alignment algorithm implemented in the High-performance Integrated Virtual Environment (HIVE), the following section describes the task of alignment and conventional methods currently used to solve it.

### Given

- There exists a set of “Reference Genomes” numbered ***1…r…N*** with sizes of ***G_r_*** and cumulative size of ***R = ΣG_r_*** bases.
- There exists a set of “Short Reads” from ***1…s…S,*** each one having a length of ***L***.

### Task

- Define an alignment as ***A***(***s***,***r***) = ***(s_1_r_1_),…(s_i_r_j_),… (s_As_r_Ar_)*** where ***(s_i_r_i_)*** signifies the correspondence between ***i***-th letter of the short sequence and ***j***-th letter of the reference sequence. ***As*** and ***Ar*** correspond to the length of the alignment with respect to the corresponding sequence or reference.
- Define a set of “Scoring Parameters” ***P*** defining the benefit and cost factors for matches, mismatches, insertions and deletions between bases ***(s_i_r_i_)*** of the short read and reference genomes
- Define an additive “Score of Alignment” as the sum of scoring factors ***SA(s,r) = Σ(P_l_)*** where **l** is chosen based on the match, mismatch, insertion or deletion of the sequence positions of ***s*** and ***r***.

### Solve

- Find an optimal alignment ***A(s,r)*** between short sequence ***s*** and reference genome ***r*** such that ***SA(s,r)*** is no smaller ***(***≥***)*** than any other ***SA(s′,r′)*** where ***s′*** is not equal ***(≠s)*** to ***s*** and/or ***r′≠r***.

(Notice that dynamic programming alignments are only optimal relative to additive scoring schemes.

If, for example, we considered a triple deletion less costly than three separate single letter deletions, the Smith Waterman algorithm, which assumes additive costs, may fail to find the best solution.).

The simplistic approach of comparing every short read position to every other genomic position, even without mismatches allowed, has a complexity of ***O(S×L×R)*** in big O notation. Such an approach has no technical value for realistic sizes of genomes (in Giga-bases ***R∼10^9^***) and high throughput sequence (HTS)-scale sequence read files (600 Giga-bases ***S×L>10^12^***) for a single run. A more realistic approach is to detect highly scoring regions of candidate positions by finding exact matches of sequence seeds of length K (K-mers), up to K = 14, either by hashing techniques[3]–[5] or by other indexed methods like full-text minute-space (FM) indexes [6], [7] as described below:

- **K-mer seed based hash indexes.** The reference genome is precompiled into a hash table where every K-mer’s occurrence is maintained in the hash container [8]. Candidate alignment positions are then detected by looping through all hash elements corresponding to each seed of length K occurring in the short read.
- **FM-index based substring search methods.** The reference genome is compiled into a compressed suffix array container using the Burrows-Wheeler transform [9]. Lookup operations are then implemented through backward iterators searching for the sequence patterns within suffix array [10].

The speed and computational complexity of detecting candidate positions are comparable for both approaches and either can be suitable depending on the exact situation. The K-mer hash compilation stage is usually much faster than FM index building, but the hash table is also significantly larger in memory than an FM-index array [11], [12]. The result of the first stage of lookups is a list of exact matches of certain lengths. The next step generally involves extension of the preliminary matches using a heuristic extension algorithm (BLAST-like) [13] or a dynamic programming method (Needleman-Wunsch [14] or Smith-Waterman [15], [16]). The key considerations of extending seed alignment to obtain the optimal alignment include:

- **Extension of the seed candidate alignment.** The detected seeds’ exact matches are extended in both directions with or without mismatches, insertions and deletions. This step is typically very fast and is of **∼O(L)** average performance.
- **Optimal alignment.** Dynamic programming techniques are usually performed in a rectangular matrix where alternative trajectories of alignments are considered concurrently. Each cell represents an alignment between two sequences at a given position. The best possible trajectory across cells is determined by cumulative alignment scores from left to right [17]. Such techniques are generally of **∼O(L^2^)** [18], [19] performance and, having square dependent memory footprint, are not cheap with respect to memory and CPU-clock.

Depending on the approach used, one may impose certain requirements on alignment algorithms to ensure the reliability of the computational results: **optimality** –demands that no better alignment is possible for the specified region of the supplied reference genome and short read; **fuzziness** – a small number of errors are acceptable within an allowed error density; **quantifiable** – each alignment can be assigned a number and score for the purpose of comparing two alternative alignments; **customizable** – behavior of alignment can be finely controlled by a set of parameters; **robustness** – small changes in parameters should lead to small changes in alignment results; **reproducible** – should arrive to the same alignment despite the stochastic nature of the algorithm’s initial detection of seeds’ exact matches.

The HIVE-hexagon aligner applies modified versions of the aforementioned approaches in conjunction with a new suffix-based approach to the removal of duplicate data and strategic sorting to optimize the alignment process: only the best candidate alignments undergo the computationally expensive stage of optimal alignment.

## Results

**Figure 1:**
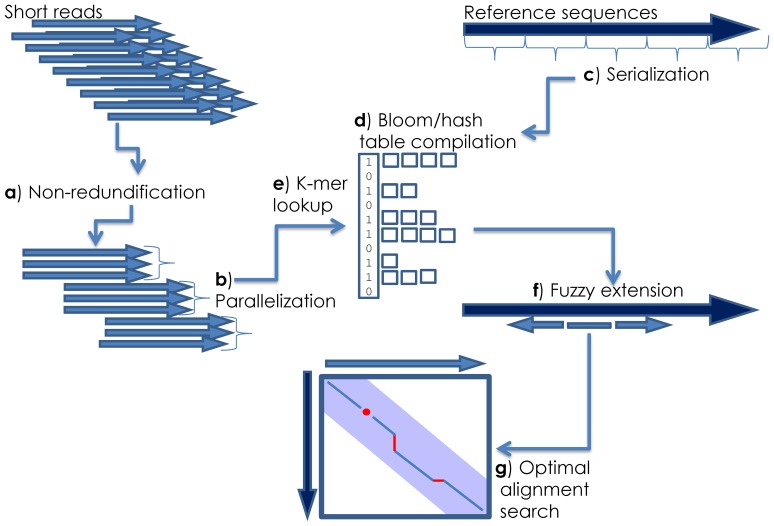
Workflow for HIVE-hexagon alignment utility. Overall alignment schema for HIVE-hexagon: short reads are non-redundified (**a**) and parallel portions (**b**) are sent to distributed cloud for computation. Reference genomes are then split into smaller pieces (**c**) and compiled into bloom/hash table (**d**). Every parallel execution thread performs a K-mer lookup against every reference sequence (**e**) then extends matches via inexact alignments (**f**) and performs a subsequent optimal alignment search on remaining candidates (**g**).

The implementation of novel and traditional approaches to the alignment task in HIVE-hexagon promotes competitive performance when compared to current industry standards. The overall alignment pipeline employed by HIVE-hexagon can be seen in Figure 1. Short read data sets are non-redundified such that only a unique copy of any given read is subjected to the alignment while appropriate counts and indexes are maintained for all such reads. Remaining reads are then sorted by sequence similarity for efficient lookup in later stages, and both reads and reference sets are distributed among the computational cloud. Parallelized reference sets are compiled into a bloom/hash table such that each read thread undergoes K-mer query against each reference, followed by extended inexact alignments for all identified matches. If an inexact alignment does not meet score requirements, self-similarity can be used to filter neighboring reads based on the implied similarity of proximal data as ensured by earlier sorting. Thus, the number of actual alignments calculated is drastically reduced. Finally, the optimal, floating diagonal adaptation of the Smith-Waterman algorithm is computed for all candidate reads that score above the specified threshold in the inexact alignment stage.

This paradigm greatly benefits HIVE-hexagon with respect to computational speed and sensitivity: the time saved allows HIVE-hexagon to take on more costly computations to achieve greater sensitivity without sacrificing overall alignment speed. To support this claim, we have performed validation and benchmarking procedures to compare the HIVE-hexagon alignment algorithm to similar software packages used in the industry. For this paper, we have chosen Bowtie and BWA for comparison since these two tools have been readily adopted for high throughput sequence alignments.

In the first category of tests we only compare algorithms without considering parallelization, computer stress factors, and performance or I/O characteristics of computations. In the second category we compare the alignment platform as a whole with competitive applications running in singular and parallel modes (where possible).

### Sample Notation

We have chosen.

- **Influenza (IZ) sample.** Multi-segmented RNA virus which is accurately represented by its H5N1 genome and the mutations and divergence of the sample were well categorized from previous Sanger sequencing data;
- ***Mycoplasma hyorhinis*** **(MH) sample.** Bacterial sample of known origin with a known set of multiple repeats determined by Sanger sequencing;
- ***Homo sapiens*** **(HS)** **whole genome sequencing sample.** Eukaryotic DNA-seq sample from Sequence Read Archive out of which artificial reads were generated from 2 reference segments (chromosomes) X and Y Human Genome v19 Build 37.3;
- **mixture of 15 viruses (VM)** out of which artificial reads are generated from 2 of the genomes Human adenovirus C (gi|9626158|ref|NC_001405.1) and Dengue virus 1 (gi|9626685|ref|NC_001477.1);
- and, similarly, a **mixture of 10 bacterial genomes (BM)** from which artificial reads are generated for 2 of the genomes Shigella dysenteriae Sd197 (gi|82775382|ref|NC_007606.1) and Streptococcus pneumonia ST556 chromosome (gi|387787130|ref|NC_017769.1).

Full information regarding mixed samples and concentrations can be found in Table S1.

### Inputs and Arguments

We have generated artificial reads from original reference sequences with no error (prefix **AR0-**), 1% error (**AR1**) and 5% error (**AR5**) or taken the original submissions produced by a sequencing hardware (**OR**). To define a consistent notation for our samples within this publication we also signify the number of reads as a suffix for the sample name. Thus, in this notation the sample AR5-IZ-1M would mean 1 million reads artificially generated from the Influenza reference with 5% random errors; similarly OR-HS-100M indicates a large, original sequence file of human origin with 100 million reads.

All HIVE-hexagon runs were performed with optimized advanced parameters including K-mer extension minimal length percent of 75, K-mer extension mismatch allowance % of 15, optimal alignment search of only identities and seed K-mer length of 14. Basic parameters were set to a minimum match length of 36 and 15 percent mismatches allowed, reporting the first match found to have the highest score of alignment. All Bowtie runs were performed with additional argument –e 600 both inside and outside of HIVE. All BWA runs were performed with additional argument –n 15 both inside and out of HIVE. These parameters were chosen to mimic the sensitivity of those tools with HIVE-hexagon as much as possible. Alignment results are reported as the percentage of unaligned reads. Detailed count information for all runs can be viewed in Table S2.

#### Proof of concept

Error free sequence files were artificially generated using the generateSeqs script (Table S3) with 1 million reads each generated from sequence data originating from influenza, mycoplasma and human samples. This test acts as a proof of concept since we expect to detect 100% of artificial error free reads when aligned to the appropriate reference. As seen in Table 1, we fully aligned all error-free reads for influenza and mycoplasma runs. A very small number of error-free human reads were left unaligned by all tools: 16 by HIVE-hexagon, 150 by Bowtie and 147 by BWA. The exclusion of some artificial reads may be due to the over-optimization of heuristic algorithms with regard to seed over-representation which can have a degrading effect on alignments. This can have a drastic impact on low-complexity read alignments as evidenced by the provided example alignments for human samples with no noise. Low-complexity sequences generate thousands of hit candidate positions, making comprehensive alignment costly and disadvantageous due to the required increase in computational time without much added benefit. Additionally, specifying a smaller seed length for the determination of candidate regions results in a larger number of positions to be considered for extension. Each one base shortening of the seed results in four times as many computations in the candidate discovery stage. Thus, the huge decrease in the amount of unmapped reads observed for more divergent samples can be explained by the gain in extra sensitivity provided by shorter seed specification.

**Table 1:** Validity and Sensitivity Comparison of Alignment Tools in Native Environments.

TEST	PURPOSE	SAMPLE	HIVE-HEXAGON	BOWTIE	BWA
**Proof of concept for** **single species**	Influenza mapping	*AR0-IZ-1M*	0.0000	0.0000	0.0000
	Mycoplasma mapping	*AR0-MH-1M*	0.0000	0.0000	0.0000
	Human mapping	*AR0-HS-1M*	0.0016	0.0150	0.0147
**Sensitivity check for** **single species**	Proof of concept	*AR0-IZ-1M*	0.0000	0.0000	0.0000
	Sensitivity level 1 check	*AR1-IZ-1M*	0.0013	0.5171	0.4930
	Sensitivity on more divergent sample	*AR5-IZ-1M*	0.4645	16.7000	21.3228
**Sensitivity check for samples with**	Mixture proof of concept for viruses: Capability to separatedifferent references	*AR0-VM-1M*	0.0000	0.0000	0.0000
**many species: Viral genomes**	Capability to separate different references with greater sensitivity	*AR1-VM-1M*	0.0310	0.4376	0.6207
	Capability to separate different/divergent references with great sensitivity	*AR5-VM-1M*	0.6079	16.7687	21.2656
**Sensitivity check for samples with**	Mixture proof of concept for bacteria: Capability to separatedifferent references	*AR0-BM-1M*	0.0000	0.0000	0.0000
**many species:** **Bacterial genomes**	Capability to separate different references with greater sensitivity	*AR1-BM-1M*	1.1817 0.0002*	0.2963	0.4078
	Capability to separate different/divergent references with great sensitivity	*AR5-BM-1M*	7.3305 0.3539*	16.2000	20.3869
**Sensitivity check for** **large genomes:**	Proof of concept for large, human genome	*AR0-HS-1M*	0.0016	0.0150	0.0147
**Human data**	Capability to separate different references with greater sensitivity	*AR1-HS-1M*	5.8383	18.8320	18.4592
	Sensitivity on more diverse sample from a large genome	*AR5-HS-1M*	15.3918	62.8203	62.4555

*with repeat and transposition search sub-algorithm on.

#### Sensitivity check for single species

Three sets of artificial reads were generated from the same influenza sample with varying degrees of error. A higher induced error rate simulates the real-life scenario of divergent sequences. Thus, this test shows if and how sensitivity varies in alignments between increasingly divergent sequences. As expected, an increasing number of alignments are missed across all tools as error or noise (indicating sequence divergence) increases. The higher sensitivity of a method for divergent sequences is critical for detection pipelines where adventitious agents present in small quantities can have adverse effects on biological products safety. An inability to detect a significant amount of sequence hits when the reference sequence is not well known can render Next-Gen based techniques useless. HIVE-hexagon has been specifically optimized to improve sensitivity and clearly outperforms both Bowtie and BWA in this respect.

#### Sensitivity check for samples with many species

As mentioned above, read sets with variable error rates were generated from select genomes within viral mixture and bacterial mixture samples. Alignment of the read files created with zero error to the entire mixture sample allows us to demonstrate HIVE-hexagon’s ability to separate multiple references within one sample. Subsequent comparison to files with error rates tests the sensitivity with which HIVE-hexagon can separate and map a query to the correct reference in a mixed file when the query has an increasing degree of divergence from its reference. Separation and mapping follows the same principle here as in the single species check such that it is increasingly difficult (and therefore more alignments are missed) as divergence increases.

The viral mixture shows HIVE-hexagon once again to surpass performance of other tools. The bacterial mixture results demonstrate a more complex mode of alignment, ultimately showing HIVE-hexagon to be more sensitive for files with or without divergence. Because the chosen bacterial mixture contains species having significant numbers of repeats, HIVE-hexagon was run both with and without a specific argument forcing careful detection of such repeats. In the repeat and transposition detection mode, HIVE-hexagon misses significantly fewer alignments while being only 10–15% slower compared to the non-repeat mode. Once again, HIVE demonstrated much higher sensitivity than Bowtie and BWA while being more time-optimal.

#### Sensitivity check for large genomes

The large genome sensitivity concept is similar to the mixture approach because of the nature of eukaryotic (human) genome references as compilations of separate reference files of various segments (chromosomes, genes, etc.). Thus, HIVE-hexagon’s ability to separate references is essential to its utility in human mappings. Furthermore, human genomes and sequence data are much larger, on average, than bacterial and viral counterparts and the stress to memory and IO in algorithms is significantly greater. Regardless, results follow the established trends with HIVE-hexagon missing fewer alignments than both Bowtie and BWA. This test showcases the viability of HIVE-hexagon as a faster and more sensitive tool for eukaryotic contiguous alignments.

### Performance/scalability Testing

#### Performance dependency on the size of the genome

The hashing of extra-large eukaryotic genomes can be a time consuming step. An FM index may take 2–3 hours to compile for BWA and Bowtie on modern x86 CPUs, but once finished, results can be maintained for application to future computations. As a native component of the HIVE system, HIVE-hexagon is required to allow users to subset and superset genomic reference sequences in an arbitrary manner during alignments and therefore cannot maintain permanent indexes of precompiled references: HIVE-hexagon recompiles the reference sequence K-mer hash tables every time a computation is initiated. Although it takes up to 8 minutes to recompile a eukaryotic size genome, this is considered an affordable tradeoff between convenience and functionality given the approximately 1–1.5 hours it takes to perform an alignment of 100 million sequences on such genomes. For smaller genomes this step takes only a few seconds and does not play a significant role in our performance considerations.

As mentioned before, low complexity regions and over-represented repeated subsequences can strongly diminish alignment algorithm performance. Large reference genomes tend to be more prone to such regions; it takes super-linearly disproportional time to map reads to such genomes compared to compact and dense genomes. With simple low complexity read filters, low complexity reference region masking and over-represented seed masking, the performance of HIVE-hexagon alignment of 100 million reads to human genome can boost from **∼**1–1.5 hours to 35–40 minutes. The sensitivity of biologically dense sequence alignments deteriorates only slightly (usually less than 0.1% in real samples) and only in long, low complexity stretches of the reference genome.

Thus, the total time consumed by HIVE-hexagon may be from 15 minutes to 1.5 hours for 100 million reads against a 3GB human genome depending on the set of parameters. Comparable and still less sensitive runs using BWA and Bowtie take about 1–1.5 hours assuming the references have been precompiled (2–3 hours).

#### Performance dependency on the coverage and the number of reads

Due to its non-redundification feature, HIVE-hexagon consistently outperforms BWA and Bowtie linearly proportional to the coverage. For example: alignment of a real dataset containing 100 million reads (out of which only 16 million were unique) to a small influenza genome (12KB) resulted in 6x time-saving (2–3 min) for HIVE-hexagon when compared to itself without utilizing the non-redundification algorithm (**∼**15 min). Similar computation against 25 pico- and entero-viruses using HIVE-hexagon took around 15–17 minutes (larger cumulative genome) for a metagenomic poli-virus environmental sample dataset of the same size where the unique count was roughly similar to the total sequence count. BWA and Bowtie both take about 20–25 minutes for the same datasets, although generally slightly less sensitive.

#### Performance dependency on the execution environment

All sequence aligners (HIVE-hexagon, BWA, Bowtie or others) implemented within HIVE infrastructure run in a parallel execution environment. The time and benchmarks compared in the two previous sections were those of the applications run from within the system. The actual running times for standalone applications can be significantly longer, and are roughly linearly proportional to the level of parallelism used to run. For example, computational alignment of a human genome of 100 million sequences which takes **∼**1–1.5 hours from within HIVE with a level of parallelism of 20 takes roughly 22–30 hours when run in a standalone, single thread mode on a comparable computer. HIVE-hexagon, specifically designed for a parallel execution environment like that provided by HIVE, additionally benefits from parallel data storage and decreased data mobility, achieving returns beyond what the algorithm itself provides.

Table S4 lists differential hit counts obtained for both Bowtie and BWA when run in their native, external environments compared to results when run inside HIVE’s parallelized environment.

### Follow-up Testing and Benchmarking

It is clear from this preliminary validation and performance testing that HIVE-hexagon has all the characteristics necessary to become an industry staple for alignments on any species. HIVE has already embarked on a number of studies and collaborations using the HIVE-hexagon aligner and other HIVE-developed tools and workflows [20]–[22], so the quality and integrity of the system and each of its tools are of the highest priority.

A number of the previously mentioned performance-enhancing ideas have been borrowed from the AceView Magic [23] alignment algorithm, and further integration of these tools is intended to continue; we therefore avoided inclusion of AceView Magic in this direct comparison but will follow-up with full benchmarking in the next publication. We plan to conduct more extensive comparisons between HIVE-hexagon and all currently available, comparable alignment algorithms within the next few months to better determine HIVE-hexagon’s respective performance and to identify any weaknesses or needs for improvement. Additionally, we intend to continue integrating other external tools directly into the native HIVE execution environment. Furthermore, we aim to use the underlying HIVE-hexagon algorithm to implement robust multiple sequence alignment, recombinant and clone discovery utilities.

## Methods

Aligning short reads to reference genomes results in a high degree of coverage, such that every genetic position is mapped by a large number of base-calls. It is not unusual to see 10,000–1,000,000× uniform coverage on shorter genomes like viruses and bacteria and 5–100× coverage on eukaryotic genomes with a lesser degree of uniformity [24]. The ratio of short read count to the number of reference positions is a heuristic measure of unique short read redundancy. Errors and noise introduced into short read sequences by sequencing chemistry or processing pipelines can reduce the actual redundancy rate by randomly introducing differences between similar DNA/RNA molecules. However, given the small systemic error rates produced by present day technologies [25]–[27], actual redundancy rates can be tens to sometimes hundreds or thousands for highly expressed regions of reference genomes. Innovative identification and usage of redundancy and cross-similarity between reads can be beneficial for bioinformatics pipelines, minimizing the storage memory footprint and bio-analytics complexity by removing repeats, and therefore allowing a higher rate of vertical compression and avoiding unnecessary repeated computations of identical/redundant reads.

Bioinformatics pipelines performing alignment and mapping computations are frequently heuristic in nature and, with memory access patterns driven by the data itself, often need to access large memory chunks randomly. For such large datasets, the “memory hungry” processes with unorganized “page hits” can create a bottleneck in computations where the vast majority of time is lost on memory pages caching into the CPU. The HIVE-hexagon algorithm proposes a novel method of linearized data organization to minimize memory usage of underlying matrixes and structures used for programming alignment algorithms, optimize memory page hits and boost algorithmic performance by taking advantage of modern CPU architecture.

The overall pipeline for the HIVE-hexagon algorithm proposed within this publication consists of a few major steps (see Figure 1):

### Non-redundification

**Figure 2:**
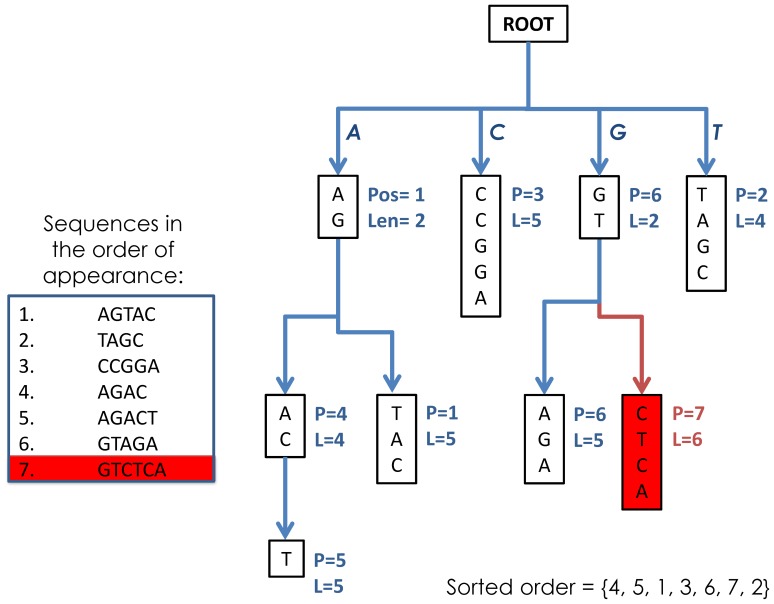
Non-redundification. This tree representation of 6 sequences is composed by linking all sequences’ tails (suffixes) to the parent nodes which represent the longest prefixes common to each branch. The tree contains all information about the 6 sequences, including position and length, at each node. Once the tree is complete, we can traverse the tree in a left maze order (always taking a left path) to obtain the sorted list of elements as: S = 4,5,1,3,6,7,2, which refers to the sorted list AGAC, AGACT, AGTAC, CCGGA, GTAGA, GTCTCA, TAGC.

The non-redundification of the sequence space is accomplished by building a sequence suffix tree [28] for text with the four letter alphabet A = a, c, g, t (see Figure 2). The use of a tree-like structure to solve sorting problems is not new in the field of bioinformatics[29]–[31]; however, previous efforts have reported problems related to the prohibitive size of suffix trees when applied to longer strings in larger quantities, increasing the string universe from hundreds to thousands or millions.

Our simple tree algorithm minimizes the number of comparisons against new sequences base-four logarithmically as they are added into the tree. Computationally, the average scenario behaves as a task of ∼O(S*log_4_L) complexity; the worst case scenario, when all sequences are identical, will require an all-to-all comparison and therefore will increase processing time to ∼O(S*L), but the corresponding storage needs are minimal. Conversely, a very diverse set of reads is computationally cheaper but has higher storage demands to accommodate the increased quantity of unique records.

Redundant sequences are discarded, but counters are associated with the unique sequences to signify that a similar sequence (in this case, an exact copy) has already been stored in the tree. Thus, each sequence in the tree at any given time is unique and, upon addition of a new sequence to the tree, it is a simple process to identify whether the sequence is a repeat or whether a new branch should be created.

The actual implementation of the algorithm has a built-in parallelization schema such that, depending on the memory limitations imposed on the algorithm from the execution environment, a decision is made to split data into 4, 16, or 4^k^ portions. Each thread will pick up sequences starting with a prefix which is the 2-na representation of its thread index in K-mer space. For example, in 16 part execution mode the portions will include the prefixes: AA…, AC…, AG…, AT…, CA…, CC…, CG…, CT…, TA…, TC…, TG…, TT… Splitting the tree parsers in this manner removes the need for a joining step where trees must be collapsed. Since no overlaps are possible in each portion and within portions, there is no need to repeat the non-redundification step and re-sort the sequences.

Another piece of valuable information stored at each sequence node is the cross-similarity coefficient which shows the degree of similarity between prefixes of consecutive sequences in the tree. This is used for explicit cross-similarity optimization of the alignment algorithm described later in this paper.

### Sorted Paths

Once all sequences are digested into the tree, it is easy to traverse the tree by visiting every node exactly once in a pre-determined order using a single traversal iteration function which operates on any given node. The function returns the node itself (which represents a sequence) or it continues the traversal through its children nodes from left to right if such nodes exist. This simple recursive procedure will, in turn, generate a final version of the file with unique and sorted sequences.

By maintaining an iterator structure on a bound, self-referenced tree this operation is of constant time access and the whole tree can be generated within **O(S)** operations where **S** is the count of non-redundant sequences.

### K-mer Hashing

**Figure 3:**
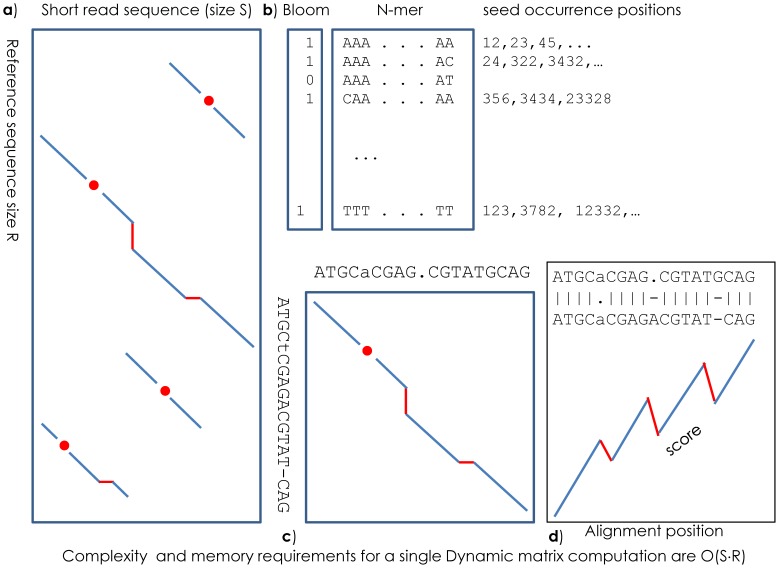
Optimal alignment search optimization schema. (**a**) Dynamic programming matrix Needleman-Wunsch or Smith-Waterman algorithms use a two dimensional rectangular matrix where cells represent the cumulative score of the alignment between short read (horizontal) and reference genome(vertical). (**b**) HIVE-hexagon maintains a bloom lookup table where each K-mer is represented only by a single bit signifying the presence or absence of that K-mer and 2-na hash table where a sequence’s binary numeric representation is used as an index. (**c**, **d**) The fuzzy extension algorithm allows accurate definition of the alignment frame and squeezes the window where the high scoring alignments might be discovered.

HIVE-hexagon compiles a dictionary of K-mer occurrences (seeds) in the reference set of genomes, where the K-mer is defined as a shorter subset of ***r_1_,…r_i_,… r_K._*** The compiled result is a hashed bucket list (Figure 3d) where each bucket represents the positions of its seed’s occurrences in a reference genome. This step has a complexity of ***O(R)***. In a genomic alphabet of four letters (A, T, G, C), the K-mer table consists of ***4^K^*** elements, each occupying so many integers in memory to hold the list of occurrence positions and the hash back reference. In a conventional hash-table implementation, one would need to store backward references to hash indexes. However, in the HIVE-hexagon implementation, the K-mers themselves are considered indexes in 2-na representation of sequence space where each nucleotide is represented by a 2-bit value (A = 00 = 0, C = 01 = 1, G = 10 = 2, T = 11 = 3). By considering sequences as indexes we remove the need to maintain the sparse hash table back-references and avoid hash collisions using an over-exaggerated hash table. There is some penalty for having to maintain significantly sparse arrays for small genomes, but the benefit outweighs the cost, especially for larger genomes where the hash table is almost fully occupied.

The list of occurrence positions in a bucket list requires at least **2**×***R*** cells of integers to refer to the index of the reference genome and to a position on the genome where the particular seed has occurred. Thus, the memory footprint for a seed-hash table is roughly in the order of **∼** ***4^K^***+**∼** ***R*** integers. Contemporary (2013) computers can realistically hold a dictionary of up to 14-mers without sacrifice to the execution environment. K-mers larger than this are typically problematic, causing too great a stress to memory and, in parallel execution environments, diminishing performance benefits of hashing by memory swapping. Additionally, long K-mers require a sacrifice in sensitivity with over 1/K **∼**7% error. HIVE-hexagon implements a double-hashing schema where lookup for K-mers larger than 14 is done by double-lookups of K-mers with ***K<14*** in consecutive continuous positions.

### Lookup Step

For every short read, HIVE-hexagon retrieves the K-mers sequentially and matches them to a seed-dictionary to obtain the list of occurrences of each particular K-mer on a reference sequence as potential candidates of alignment position. A genome of size ***R*** has in average ***R/4^K^*** occurrences of candidates for every K-mer. Increasing ***K*** results in fewer candidate positions where each has a higher chance of being a true alignment, thus increasing the speed of computations. However, an increase in ***K*** also has the potential of increasing the footprint of the memory as **∼** ***4^K^***+**∼** ***R***. For ***L*** positions of the sequence there are ***L-K*** (usually ***K<< L***) positions to be looked up using the dictionary. Thus, the complexity of lookup step is ***O(S×L)*** and the memory footprint measures as **∼** ***R/4^K^*** **×S×L** with proportionality coefficient dependent on the relatedness of the reference sequence and the number of successful alignments.

A significant number of hash lookups are misses, and from the perspective of the CPU the lookup results in a memory page load on a random access basis and is therefore costly and to be avoided. HIVE-hexagon maintains a bloom lookup table (Figure 3d) [32] where each K-mer is represented by only a single bit, signifying the presence or absence of that K-mer in the table. Due to its more compact size, the lookups from the bloom table are much less costly from a paging viewpoint and result in significant time-saving. The bloom table itself occupies a single bit for each hash element instead of full size 64 bit integer; therefore, the additional cost of the bloom implementation is only **∼**1–2% more memory compared to the original bloom-less variant of the algorithm.

The HIVE-hexagon implementation of the lookup table ignores seeds which are overexpressed as defined by a count greater than a given threshold: overexpressed seeds are usually present in low complexity regions in eukaryotic genomes and, although not always, frequently do not represent biological relevance. However, if the actual biological genomic region does have such overexpressed seeds adjacent to those with normal level of expression, the alignment algorithm will be able to capture the exact seed match in subsequence k-mers and then extend as much as needed regardless of the seed’s expression level. If the entire region is made of overexpressed seeds, HIVE-hexagon will exclude alignments above a certain number of findings.

HIVE-hexagon can maintain seeds of K-mers on every genomic position, but it can also skip positions based on expression levels and preset parameters. This decreases the memory required for bucket list storage and decreases the pool of candidate positions without a great sacrifice to sensitivity for cases where the reference is well known. In viruses and bacteria, divergence can achieve large values and so this technique may decrease the sensitivity due to the fact that an exactly-matched seed has been skipped. For eukaryotic genomes, however, the reference tends to be more stable, so we frequently observe continuous exact matches longer than that of the K-mer size chosen for HIVE-hexagon.

### Bracketing and Fuzzy Extension

Once K-mer seeding has detected a candidate region, HIVE-hexagon determines the frame of possible alignment windows around the exact seed match positions. The presence of a short exact match only hints at the potential alignment: the actual alignment must be computed by other means. Extension methodologies (like that in BLAST) do not need an exact reference frame of alignment, but dynamic programming methods require a strictly defined matrix where the optimal alignment will be computed. Underestimation of the frame may result in loss of sensitivity whereas overestimation may result in vertical extension (see Figure 3a) of the matrix, slowing down computations by increasing the absolute size of the matrix, and therefore the memory and the number of computations to be performed.

HIVE-hexagon implements both double-sided extension of the seed and a dynamic programming matrix. The fuzzy extension algorithm is similar to cost-based dynamic programming methods except that it runs in a very small floating window along the bidirectional extension front using integer arithmetic. This approach not only allows more accurate definition of the alignment frame (Figure 3c), but also filters a significant number of accidental K-mer hits. The number and the density of mismatches, insertions and deletions allowed during extension are customizable and, by default, correlated with relevant parameters for the optimal alignment algorithm used downstream.

### Optimal Alignment

**Figure 4:**
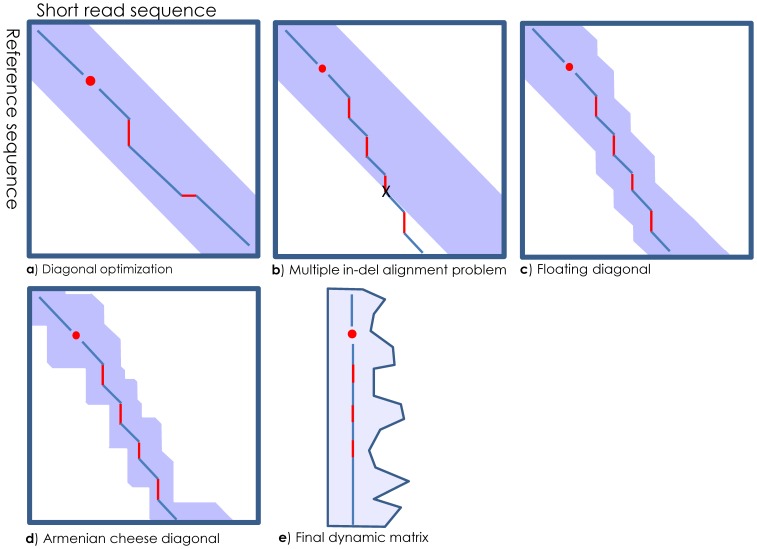
Dynamic programing matrix linearization schema. HIVE-hexagon implements a floating diagonal approach where the diagonal of the computation is maintained along the two sides of the current highest scoring path of the matrix. (**a**) We assume the optimal alignment will be along the diagonal. (**b**) Multiple insertions or deletions can result in the optimal path traversing outside the defined diagonal belt. (**c**) By defining a constant width to the diagonal and pre-computing cell scores line by line, the limits of the remaining diagonal matrix can float along with the likely optimal scoring path. (**d**) Furthermore, pre-computation of cells in the diagonal line by line allows the exclusion of regions which cannot possibly contain the highest scoring path. (**e**) The resulting minimized, dynamic diagonal matrix is linearized to further simplify the process and minimize the memory footprint required.

Dynamic programming methods of alignment [33] matrix evaluation usually include the computation of all alternative matches, mismatches, insertions and deletions (Figures 3 and 4). Each cell’s value is computed as the best score from all alternate trajectories leading to that cell either as a match/mismatch (diagonal), insertion (vertical) or deletion (horizontal). All possible cumulative scores are computed across those trajectories and the highest value ***SA(s,r) = Σ(P_k_)*** is reported as a potential alignment score along with the trajectory ***A(s,r)*** leading to it. The usual strategy involves computing the dynamic matrix values and backward pointers from the top left corner down to the bottom right corner. Backward pointers are then propagated in the opposite direction starting from the maximal score position to re-identify the trajectory which generated best local or global alignment.

The first, most obvious level of NW/SW optimization implemented in HIVE-hexagon is to avoid computation of the whole matrix and concentrate only on the diagonal region (Figure 4a) where the expected alignment usually lies because the extension algorithm applied in HIVE-hexagon ensures the accuracy of the frame positioning. Using a diagonal of constant width allows translation of computational complexity of ***O(L×G_r_)*** into ***O(L×w)*** where ***w*** is the constant width of the diagonal and does not scale with the size of the selected reference segment.

Alignments with multiple insertions or deletions can be problematic for such optimizations. Although not usually an issue for short read alignments, a significant number of multiple in-dels is a critical problem (Figure 4b) for longer contig alignments or mutual alignments of reference genes. Unlike existing analogues [34], [35], HIVE-hexagon allows the diagonal to float along the two sides of the highest scoring path (Figure 4c), thus allowing the generation of longer, multiple in-del-containing alignments to take advantage of the dynamic matrices diagonalization method.

To even further reduce the amount of computations in memory, HIVE-hexagon maintains a variable width diagonal (Figure 4d). Cells located at a greater distance from the optimal diagonal have an associated cost of insertions and deletions. Thus, alignments involving these cells will have a limit to the maximum possible score and minimal number of insertions, deletions and mismatches. Using this information, HIVE-hexagon estimates each particular cell’s potential to generate a successful alignment within required thresholds. If the maximum possible score implies no potential, that position will be ignored and no memory will be allocated for it nor will computations be performed for trajectories through that cell. For this reason, the diagonal itself has “holes”: black, ignored spots, hence the association with a particular kind of Armenian string cheese which looks like a string with variable thickness along its profile with possible holes on the sides.

Additional significant optimization implemented in HIVE-hexagon is the linearization of the final dynamic matrix. The benefit of storing a linear matrix **∼** ***w×L*** instead of the rectangular matrix ***L×G_r_*** where memory requirements are tens or hundreds of times larger is significant. In a real parallel execution environment where hundreds of processes compete for memory pages, such optimization has a huge potential of improving the actual execution speed.

### Additional Features Contributing to Competitive HIVE-hexagon Performance

The alignment further benefits from the consideration and implementation of the following:

- **Alignment of unique sequences only.** By aligning every redundant sequence only once and maintaining the count of redundancies, HIVE-hexagon already achieves significant improvements in computational speed without additional algorithmic modifications. The speed benefit resultant from non-redundification is above linear due to the decrease in stress to memory pages, caching and swapping.
- **Sorting and implicit cross-similarity.** Further lazy optimization is achieved implicitly due to cross-similarity and sorting of the short reads computed during non-redundification of short read sequences.

Seed lookup involves reading particular locations from the bloom table, from the hash table, and therefore from the computer’s memory. Modern CPUs optimize memory-reading by double-layer caching of memory pages. Random access to memory is usually slower than sequential access with additional hits to pages already in the cache.

In an attempt to align the first sequence, the CPU is forced to load a certain number of pages (containing seeds, reference genome, etc.) into the cache. If the next sequence is very dissimilar to the first, the pages loaded are different for each, thus resulting in cache saturation with potential of dumping memory pages. However, if the sequences are very similar, the seeds hit the same set of positions in memory and the CPU does not need to reload new pages. HIVE-hexagon greatly benefits from the implicit optimization of short read sorting prior to alignment. We see up to 2-fold improvement of the speed solely due to sorting.

- **Explicit cross-similarity.** Even deeper optimization can be achieved explicitly by considering cross-similarity among the short reads. If two sequences are similar up to a certain number of characters, the alignments of those to the reference genome are expected to be similar.

**Figure 5:**
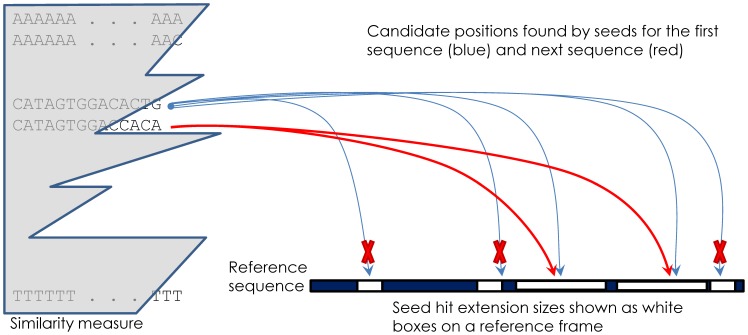
Cross-similarity decreases the pool of optimal alignment candidates. In this example, the first sequence CATAGTGGACACTG has generated 5 possible hit candidate positions (blue arrows), but only three of those have failed to extend or align long enough (marked by **X**). The next sequence, CATAGTGGACCACA, having a significant length of prefix similar to the prior one, does not need to consider all candidate positions, and can specifically exclude from consideration those for which the extension attempt failed with a wide margin of error for the prior, similar sequence.

Let us assume the first sequence has hit the K-mer table in a large number of candidate positions, out of which only a few have passed the extension step filter (described above) and even fewer have resulted in a real alignment for a given score threshold. The following sequence is nearly identical to the first and can therefore skip all candidate positions where the extension attempt for the first failed with a wide margin of error. Since HIVE maintains all self-similarities between consecutive sequences, it is possible to quantitatively predict if the best potential alignment score between a sequence and the candidate position is within the required error threshold. Thus, only a few successful hits or near-successful mis-hit positions are typically considered for real optimal alignment search (Figure 5).

The pattern of cross-similarity in non-redundant sequence files is oscillatory, frequently in a zig-zag pattern where the “zigs” (horizontals) are changes in a base early in the sequence and “zags” (diagonals) are changes at the end of the sequences. Given the inexact nature of the alignments, each consecutive sequence has to be considered in more places than assumed by the cross-similarity, so the efficiency of zig-zag is not a full 100%.

We observe up to 2–4x fold performance boost depending on the parameters defined in the alignment algorithm, assuming the choice of the reference is accurate. For these cases we notice almost no sacrifice to sensitivity (<0.01%), but for cases with reference sequence further from the subject we saw some degradation (1–2%) of sensitivity with strong usage of cross-similarity.

This parameter is optional in HIVE-hexagon and is not recommended to be used in its current stage for references distant from the subject, or for references containing multiple repeats and transpositions. We do recommend using this option to benefit from significant improvements of the speed for more accurate references. We expect further development to take place on this subject.

## Discussion

In this article, we have discussed the challenges associated with NGS data analysis, with special emphasis on alignment, and provided improvised and novel approaches to overcoming these challenges. Specifically, we have introduced new and significantly improved algorithms developed by HIVE which greatly reduce the memory footprint required by alignment of NGS data and, therefore, decrease the overall time needed for the alignment process.

A great quality of these HIVE algorithms is that the decrease in computational cost, memory requirement and time for processing is not accompanied by a sacrifice in the quality of the approach or the results. In fact, being native to the massively parallelized HIVE environment, the overall speed increase afforded by the infrastructure alone allows these algorithms to perform at a higher sensitivity than other industry algorithms of similar functionality while still outperforming in terms of time required for the computation.

Experiments have shown HIVE-hexagon is more sensitive and faster than current industry standard alignment algorithms due to scalability, high parallelizability, non-redundification, dynamic matrix linearization and implicit and explicit cross-similarity usage. There is already a great deal of interest surrounding HIVE-hexagon, and HIVE plans to continue developing this and other new tools to further promote the advancement of NGS technologies and the larger field of genomics.

## Supporting Information

Table S1 Genomes, Mixes & Conc. Lists the components and accession IDs of all sequences used in the validation and testing section of the paper.
(XLSX)

Table S2 All Run Counts. A detailed spreadsheet containing all counts of alignments (and unaligned sequences) used to summarize the validation and testing section.
(XLSX)

Table S3 generateSeqs. Provides the text of a short script written to generate the random subset of reads used in the validation and testing.
(XLSX)

Table S4 Externalvs.HIVE Bowtie&BWA. Shows the compared alignment counts of external tools when run both inside the HIVE environment and in their native external environments.
(XLSX)

Table S5 Access Data in HIVE. Provides access information and instruction for reviewers and other interested individuals to see and replicate the results presented herein.
(XLSX)
